# Conventional or Organic? Motives and Trends in Polish Vegetable Consumption

**DOI:** 10.3390/ijerph19084667

**Published:** 2022-04-12

**Authors:** Katarzyna Mazur-Włodarczyk, Agnieszka Gruszecka-Kosowska

**Affiliations:** 1 Faculty of Economics and Management, Department of Enterprise Management, E-Business and Electronic Economy, Opole University of Technology, E-Business and Electronic Economy, 7 Luboszycka St, 45-036 Opole, Poland; 2 Department of Environmental Protection, Faculty of Geology, Geophysics, and Environmental Protection, AGH University of Science and Technology, al. A. Mickiewicza 30, 30-059 Krakow, Poland; agnieszka.gruszecka@agh.edu.pl

**Keywords:** vegetable intake, eating habits, consumer trust, sustainable consumption, harmonization, food labeling

## Abstract

Vegetables constitute a major component of human food security. They are the main sources of essential nutrients including antioxidants, natural dyes, minerals, and vitamins. Eating habit issues related to the consumption of vegetables are gaining importance within the context of a healthy lifestyle, longevity, and physical fitness. Additionally, food quality is of primary importance, and so-called eco-food (defined as food as natural as possible, without fertilizers, pesticides, or preservatives) seems to be the most popular world-trend in healthy nutrition. Keeping these ideas in focus, research on vegetable consumption in Poland in the context of conventional or organic production was performed using online questionnaire surveys. The results revealed that the rate of vegetable consumption depended primarily on economic status, except for the potato, which was a staple cutting across all economic strata. Among the 108 analyzed respondents, 74% bought vegetables from certified organic farms. However, 59% bought organic vegetables “rarely” or “sometimes”, and only 15% “often”. Next, respondents chose to buy vegetables from fresh food markets (45%) and in local shops (41%). About 20% of the respondents acquired vegetables from their own farms. Among the reasons for choosing vegetables from certified organic farms, respondents mentioned in decreasing order: “desire for proper nutrition” (30%), “thinking that organic vegetables are healthier” (28%), and “organic vegetables are generally better” (7%).

## 1. Introduction

Vegetables are undeniably an important element of the food basket, both in terms of their nutritional and health-promoting values. The importance of a balanced diet in disease prevention is no longer questioned. Apart from the dietary benefits, the other major aspect of vegetable consumption is its sustainability. A general definition is that sustainable development is designed to meet the needs of current generations without damaging natural resources so that future generations can also provide for their needs. In this context, sustainable consumption is thus referred to as a derivative of sustainable development as it aims at increasing awareness and actions to protect present and future generations [1].

The sustainability of vegetables is not only reflected in a balanced diet, but also in other environmental aspects that affect the standard of living of present and future generations such as biodiversity, protection of ecosystems, cultural acceptance, accessibility, economic fairness, affordability, and the optimization of natural and human resources [2]. Vegetables have a lower carbon footprint than most other foods [3], meaning that their production has less negative impact on the environment than, for example, meat production (this also applies to the amount of water consumption, generation of pollutants, and methane [4]). In terms of sustainable development, the production of farm crops is among the world’s greatest challenges. Because nutrient rich soil is essential to healthy plant life, ensuring its quality by reducing or eliminating soil pollution and promoting a more organic approach to agriculture seems to be among the most important agricultural sustainability goals [5].

Vegetables are defined as herbaceous plants that are in whole or partially consumed by humans. It is recommended to eat (along with fruit) at least 400 g of vegetables per day (five servings) for a healthy diet [6]. This is not only due to the nutritional properties of vegetables, but also their positive influence on an individual’s psychophysical condition. Vegetables are essential to a balanced diet because they are a key source of nutraceuticals such as antioxidants, natural colors, minerals, and vitamins [7]. Their consumption affects the condition of the digestive tract, reduces the risk of a heart attack, prevents certain types of cancer [8,9], diabetes [10], obesity [11], and enhances proper cognitive functions [12]. Eating habits related to the consumption of vegetables are particularly important in times of intense lifestyle changes (e.g., related to the COVID-19 pandemic, which has significantly reduced physical activity and changed the structure of purchases carrying the additional risk of weight gain [13] and an overall deterioration of health). Vegetable deficiency in a diet may also affect the quality and duration of sleep [14] and cause symptoms of depression [15]. Even though the portions suggested by the World Health Organization (WHO) apply to both fruit and vegetables, it is worth consuming more vegetables (due to the lower content of simple sugars). In addition, due to the different content of nutrients, it is also important to consume a variety of vegetables in terms of their color [16].

However, the average global consumption of vegetables is lower than recommended by the WHO [17,18,19,20,21], and current eating habits are not always fully consistent with a recommended healthy and balanced diet [22], which is beneficial to health in terms of food and nutritional security. The WHO recommends eating >400 g of vegetables and fruits each day and half of this quantity should consist of vegetables [23]. According to [24], the statistics, Poles consumed 7.7 kg of vegetables in 2020. Regarding the above, on average, one tenth of the recommended vegetable amount is consumed in Poland.

The low consumption of fruit and vegetables increases with age and decreases with income [25] and depends on the gender—women consume more vegetables than men [18]. Other influencing factors include average monthly incomes per capita in household, place of residence (city, village), region of residence (voivodship/province), socio-economic groups (employees, farmers, self-employed, older-age and disability pensioners), the number of people in the household [26], health condition, culture, and community influence [27] as well as the sensory components—the smell, taste, appearance, and texture of food products [28]. It is also related to food security [29].

The amount of vegetable consumption per person in a household is used as one of the indicators of sustainable development [30]. According to Statistics Poland [31], this index is calculated for vegetables such as cabbage, cauliflower, tomatoes, cucumbers, other vegetables with edible fruits, carrots, beets, onions, other root, and tuber vegetables, vegetable mixtures, legume seeds, mushrooms, mushroom, and potato preserves. Among the different factors that determine sustainable consumption patterns such as the number of passenger cars per capita or the level of electricity consumption in households per capita, individuals vegetable consumption is used as an indicator [30] (pp. 18, 70).

**Figure 1:**
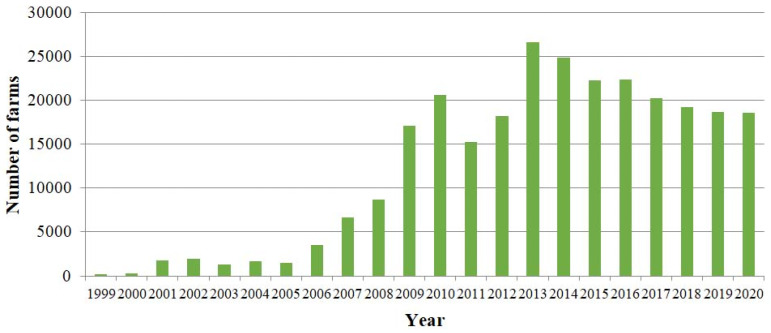
Number of organic farms in Poland in the years 1999–2020. Source: Own study based on the Inspection of Purchase and Processing of Agricultural Products [31] (p. 7); [32] (p. 9); [33] (p. 10); IJHARS [34] (p. 5); [35] (p. 7); [36] (pp. 11, 12); [37] (pp. 13, 14); [38] (pp. 24, 38); [39] (pp. 26, 42); [40] (pp. 23, 36); [41] (p. 33); [42] (p. 32); [43] (p. 31).

**Figure 2:**
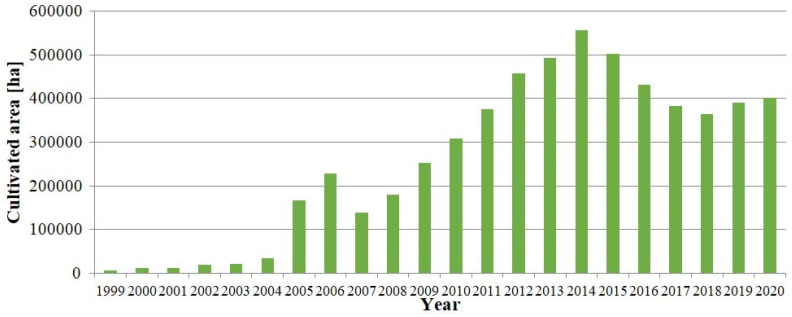
Area of organic farming [ha] in Poland in the years 1999–2020. Source: Own study based on the Inspection of Purchase and Processing of Agricultural Products [31] (pp. 8, 20); [32] (p. 9); [33] (p. 8); IJHARS [34] (p. 8); [35] (p. 8); [36] (p. 15); [37] (pp. 19, 20); [38] (p. 40); [39] (p. 43); [40] (p. 37), [41] (pp. 50, 51); [42] (pp. 48, 49); [43] (pp. 45, 46).

In Poland, the development of the organic food industry has been progressing dynamically since 2009. This tendency resulted from both the increased number of organic farms in the years 2009–2020 (Figure 1) as well as increasing the area of organic farming carried out by these farms (Figure 2). This may be the result of globalization and the adoption of certain trends, the increasing wealth of Polish society as well as the average consumer paying more attention to their overall health and well-being.

In 2019, the Polish Center for Accreditation was authorized to accredit certification bodies, while the Control Bodies of Organic Farming were authorized to issue, inspect, and withdraw certificates in organic farming. At that time, 13 certification bodies were entitled to authorize organic farms, organic processing plants, and their subcontractors. In 2019, there were 20,000 Polish organic producers. The largest class among them were farmers or growers (92.5%). Other members included in the producer class were certified organic seed producers and producers of essential seed production support products as well as natural harvesting processes and beekeeping. Organic producers were located in following administrative provinces of Poland in decreasing order: Warmińsko-Mazurskie (16.2%), Podlaskie (14.4%), Mazowieckie (13.3%), Zachodniopomorskie (10.5%), Lubelskie (10.3%), Podkarpackie (5.4%), Wielkopolskie (4.6%), Lubuskie (4.4%), Małopolskie (4.2%), Dolnośląskie (3.8%), Świętokrzyskie (3.4%), Łódzkie and Pomorskie (3.0% each), Kujawsko-Pomorskie (2.2%), Śląskie (1%), and Opolskie (0.4%) [43]. However, it is rather difficult to find information about the sellers of organic vegetables as well as comparative data presenting their advantages.

**Figure 3:**
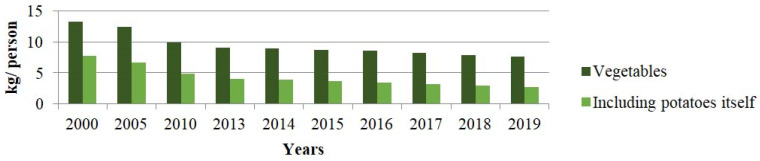
Average monthly consumption of vegetables in Poland (kilogram/person) in the years 2000–2019 [26] (pp. 183, 336).

Almost one fifth of the 250 species of vegetable plants are cultivated in Poland [44]. Although Poland has been recognized as a leader in the production and export of many vegetables (including white cabbage, mushrooms, carrots, onions, tomatoes, beets, and cucumbers) [44], their consumption has not increased in comparison to other European countries. In fact, in 2019, a decrease in the consumption of vegetables in Poland continued, a detailed list is presented in Figure 3. The average monthly consumption per person was: 2.75 kg of potatoes, 0.36 kg of cabbage, 0.79 kg of tomatoes, 0.47 cucumbers, 0.16 kg of beets, and 0.41 kg of carrots [26] (p. 184).

According to the data reported by Statistics Poland [26] (pp. 141, 213), the level of vegetable consumption (excluding potatoes) depends on a household’s income. In terms of Polish socio-economic groups, vegetable consumption is the highest among farmers, retirees, pensioners, and marriages either without children or with one dependent child [26]. Moreover, as part of the monthly funds allocated by households on consumable goods per person (that is, PLN39.34–US$9.9) on average PLN22.02 (US$5.5) was allocated to fresh and frozen vegetables. Respectively, PLN6.12 (US$1.5) was spent on potatoes, PLN1.44 (US$0.4) on frozen vegetables and mushrooms, and PLN9.70 (US$2.4) on other vegetable preserves.

In 2019, the highest amounts of vegetables were consumed by the inhabitants of the Świętokrzyskie Province, who on average consumed nine km/person per month. It was 1.4 kg more than in Warmińsko-Mazurskie Province, where the consumption was the lowest in the country. Among the provinces with the lowest level of vegetable consumption, the Wielkopolskie Province should also be mentioned with the result of 6.82 km/person [26] (p. 229). The available statistical data do not show the quantity of vegetables consumed in single servings (consumed at once).

Previous studies [26,27,28] have shown the increased consumption of vegetable preserves, along with a decreased consumption of fresh vegetables and the replacing of domestic vegetables with imported ones. However, an increased diversity of consumed vegetables also became evident [45]. When limiting the research on the balanced diet of Poles to children and young adults, it is worth noticing that the insufficient consumption of vegetables among the youngest has been influenced by parents as well as the availability, quantity, and variety of vegetables consumed at home [46]. Moreover, younger children (at the age of 3) eat vegetables more often than older children, and the frequency of consumption of vegetable juices decreases with age [47]. The decreasing vegetable consumption also applies to students, among whom the frequency of vegetable consumption is also far from the nutritional recommendations [48]. Similarly, among the oldest Poles, it was also observed that they did not consume vegetables in the recommended quantity of five servings a day [49].

At dinner, Poles consume vegetables that are raw, rather than as part of a dish [50]. According to the research conducted by Kawalec and Pawlas [51], vegetables are not a popular breakfast ingredient among Polish children—one third of respondents had never eaten vegetables for breakfast. On the other hand, the study conducted by Skolmowska et al. [52] among students of upper secondary schools showed that this group of consumers could be divided into three subgroups: ‘low-preferring’, ‘hedonists’, and ‘high-preferring’, and only the last of them declared consuming at least one portion of vegetables or a salad along with the evening meal.

The low consumption of vegetables in Poland may be caused by increased prices, changing consumption habits (including the use of semi-and processed food), less free time due to more time spent at work [53], and lack of knowledge. According to the data provided by the National Union of Fruit and Vegetable Groups of Producers [50], despite being aware of the benefits of vegetable consumption, three quarters of Poles do not know how many vegetables they should eat, while one third believe they eat as many vegetables as they should. However, awareness is not always enough to implement sustainable practices. According to the study conducted by Suliga et al. [54], despite their extensive knowledge of food and nutrition, Polish students consume less vegetables than German students. Therefore, the knowledge itself is not always reflected in the diet. The availability of vegetables and the way they are served (e.g., portion size) is also important.

Based on the above, the aim of this research was to analyze the preferences of Polish consumers regarding the purchase and eating habits of vegetables based on consumer attitude toward vegetables grown in a traditional and so-called ‘organic’ way. Considering that the centrally collected statistical data do not show detailed consumption tendencies for individual edible plants, this preliminary research aimed at increasing the awareness of Poles on sustainable vegetable production, the availability of ecologically certified products, the motives for choosing or rejecting certified products, and eating habits in the context of slow-food and eco-food movements.

## 2. Materials and Methods

To determine the preferences of Poles in consuming vegetables, a survey was carried out based on a questionnaire designed by the authors.

The following vegetables grown in Poland and consumed by respondents were included in the survey (Table 1): aubergine, broccoli, beetroot (including beet greens), broad beans, onion (white, red, shallots), horseradish, zucchini/squash, garlic, pumpkin, beans (white, red, Hansel, Pearl, Bomba), peas, kohlrabi, cauliflower, cabbage (white, red, Italian, Beijing, Brussel sprouts), corn (cob), lovage, carrots, cucumbers (English, pickling), pepper (ground, sweet, hot), parsley (tops, root), tomato, leek, rhubarb, radish, arugula, lettuce (ice, butter, Romaine, lamb’s lettuce, oak leaves), celery (root, celery), chives, asparagus, spinach, and potatoes, without identifying which part of the plant, except when one-time consumption depends on the specificity of a given vegetable, and thus its useful part (this applies to beetroot, horseradish, corn, parsley, and celery).

The survey was conducted in 2017 (between February and November) using the Interankiety.pl platform (in a digital form). The survey was conducted using non-probability, exponential non-discriminative snowball sampling [55] where existing respondents recruited further subjects from among their acquaintances. The sampling was virtual as the survey was prepared in digital form, and mainly scientific (e.g., ResearchGate or LinkedIn) and social (e.g., Meta) networks were used for the dissemination of the link to the survey.

The questionnaire consisted of 92 questions divided into three parts. The first part was a general section in which the respondents were asked five questions regarding where they bought vegetables (possible answers: “market”, “greengrocer’s”, “supermarket”, “neighborhood shop”, “health-food store”, “self-cultivated”, “not applicable”), if they bought certified organic vegetables (possible answers: “always”, “nearly always”, “often”, “rarely”, “almost never”, “never”, “don’t know”, “other”), what their reasons were for buying (possible answers: “they are better than traditional ones”, “it is trendy”, “they are healthier than traditional ones”, “the desire for healthy eating”, “I can afford it”, “not applicable”) or for not buying (“they are too expensive”, they are not healthier than traditional ones”, “I am not sure what is their eco-friendliness”, ”no possibility of purchasing in the place of residence”, “they are not better than traditional ones”, “it does not matter to me”, “I do not see any difference compared to traditional ones”, “not applicable”) certified vegetables. In this part, respondents were asked to choose the most suitable answer from the given propositions. In the case of any question in which the respondents did not find the relevant answer, they could choose the option “other” and provide their own answer.

The second part of the questionnaire consisted of 78 questions in which respondents were asked how often and in which quantity at one time they consumed 36 types of vegetables (Table 1) that were grown and commonly consumed in Poland. Participants were asked to estimate how much food they consumed in a serving, and two different ways in which to do so were proposed: one more specific (in grams) and another that was broader (portion size). In this section, respondents were asked to choose the most suitable answer from the given propositions.

Regarding frequency of the particular vegetable consumption, the possible answers were as follows: “more than three times a day”, “three times a day”, “twice a day”, “once a day”, “six times a week”, “five times a week”, “four times a week”, “three times a week”, “twice a week”, “once a week”, “several times a month”, “dozen times a year”, “several times a year”, and “I do not eat it at all”. Regarding the quantity of consumed vegetables, the following units were used: leaves, stalks, twigs, a bunch, florets, cloves, piece, a pinch, a handful, a glass, slices, and their relevant multiplications depending on the type of the vegetable (e.g., three slices or half of glass). These informal units of consumption were prepared for those respondents who preferred visual units. In parallel, the mass of vegetables in g (and its multiplication) was given for those respondents who preferred to define consumption rate in the mass units. Respondents were allowed to choose either visual volume unit or mass unit. Again, if the respondents did not find the relevant answer regarding frequency or quantity, they could choose the option “other”, and provide their own answer.

The third part of the questionnaire consisted of sociodemographic questions. Respondents were asked about their gender, age, educational level, marital status, region (voivodeship), area of residence regarding the number of inhabitants, number of people in the household, and indicative net income. Details regarding possible answers in particular sections of the sociodemographic part of the survey are given together with the results in Table 2.

As our survey was long and tedious, for further investigations, only complete questionnaires were processed for further investigation. This means that only questionnaires that provided answers to all questions in all three parts of the survey (“other” or “refuse to answer” were considered as a given answer) were valid. Ultimately, 108 complete questionnaires qualified for the analysis. All questions were answered by adult Poles, who lived in Poland, declared eating vegetables, and were mainly responsible for supplying their households with vegetables. Furthermore, as the aim in our survey was to obtain only one—the most suitable answer—from each respondent, multiple choice answers in the questionnaire were not possible.

## 3. Results

### 3.1. The Characteristics of the Research Group

The majority of respondents were women (73%). In terms of marital status, over half of the respondents were married or in a long-term relationship (59%). The second largest group were single (31%). Most of the respondents were aged between 21 and 50 years (75%). The largest group of respondents were highly educated (73%). The largest group of respondents (92%) inhabited urban areas including Opole Province (27%), Małopolskie Province (24%), Łódzkie Province (20%), and Dolnośląskie Province (14%). The households of the respondents were usually composed of two people (47%), four people (19%), one person (16%), and three people (15%). The declared amount of net earnings of the respondents usually ranged between PLN1000 and PLN3000 (US$251.7–$754.5; 52%). Detailed sociological and demographic data of the studied group are presented in Table 2.

### 3.2. The Characteristics of the Supply Sources of Vegetables

**Figure 4:**
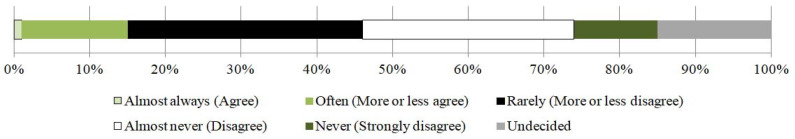
Respondents who bought organic vegetables [%].

The respondents usually bought vegetables at a supermarket (66%), at a greengrocer’s (53%), at the market (45%), and at the local store (41%). Over one fifth of the respondents grew vegetables on their own (22%). Respondents in relationships were shopping in local stores more often than single participants. Only about one tenth of respondents (12%) bought vegetables at health food stores. Among the respondents, 74% declared they bought certified organic vegetables. Nevertheless, 59% of those who bought them did so rarely or occasionally, and only 15% bought them often (Figure 4).

**Figure 5:**
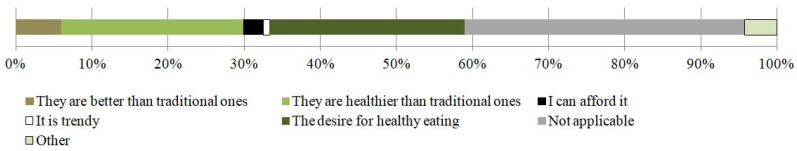
Reasons for purchasing organic vegetables [%].

The main reasons for buying organic vegetables were the willingness to eat healthy (30%) and the belief that organic vegetables are healthier (28%) or generally better (7%). Among the answers categorized as ‘other’, respondents indicated taking care of their children and their diet (3%), if “[...] these are the only vegetables available in the store”, or “if both organic and non-organic vegetables have the same price, I buy organic vegetables” (1%), and paying attention to the ecological aspect of the cultivation being ‘less harmful to the environment’ (1%). Details are presented in Figure 5.

### 3.3. Consumers’ Reason for Purchasing Organic Vegetables

**Figure 6:**
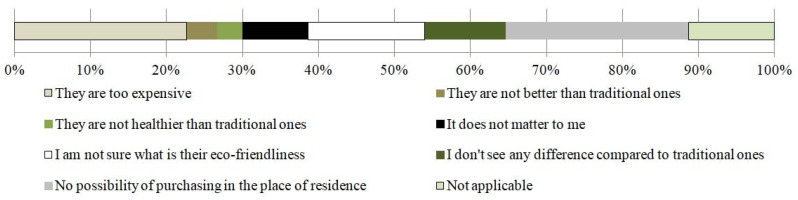
Reasons for not purchasing organic vegetables [%].

Respondents who did not buy organic vegetables referred to the inability to buy them close to the respondents’ residence (36%) and their price (34%). In addition, in the opinion of the respondents, the actual benefits of organic vegetables are questionable in terms of understanding the essential issues relating to ecology (23%) and not understanding the differences between organic and non-organic vegetables (16%). According to those who did not buy organic vegetables (category ‘other’), “certificates are often not reliable as they are awarded by a mutual adoration society” (1%). Detailed answers are presented in Figure 6.

### 3.4. The Characteristics of the Consumption Trends of Vegetables

In terms of vegetables eaten daily, the respondents most frequently declared: onions (22% of the respondents), tomatoes (21%), and carrots (10%). On a weekly basis (i.e., eating vegetables one to six times a week), the respondents also consumed potatoes (70%), cucumbers and spinach (56% each), garlic (51%), lettuce (49%), corn (46%), kohlrabi (43%), pumpkin (42%), chives (32%), rhubarb and broccoli (30% each), and broccoli (30%). On the other hand, white radish and asparagus were eaten the least. Detailed answers are presented in Table 3. Among the vegetables not grown in Poland but consumed by respondents, individual participants mentioned sweet potatoes, bamboo shoots, and lemongrass. According to the respondents, these plants were consumed by them several times a month.

**Figure 7:**
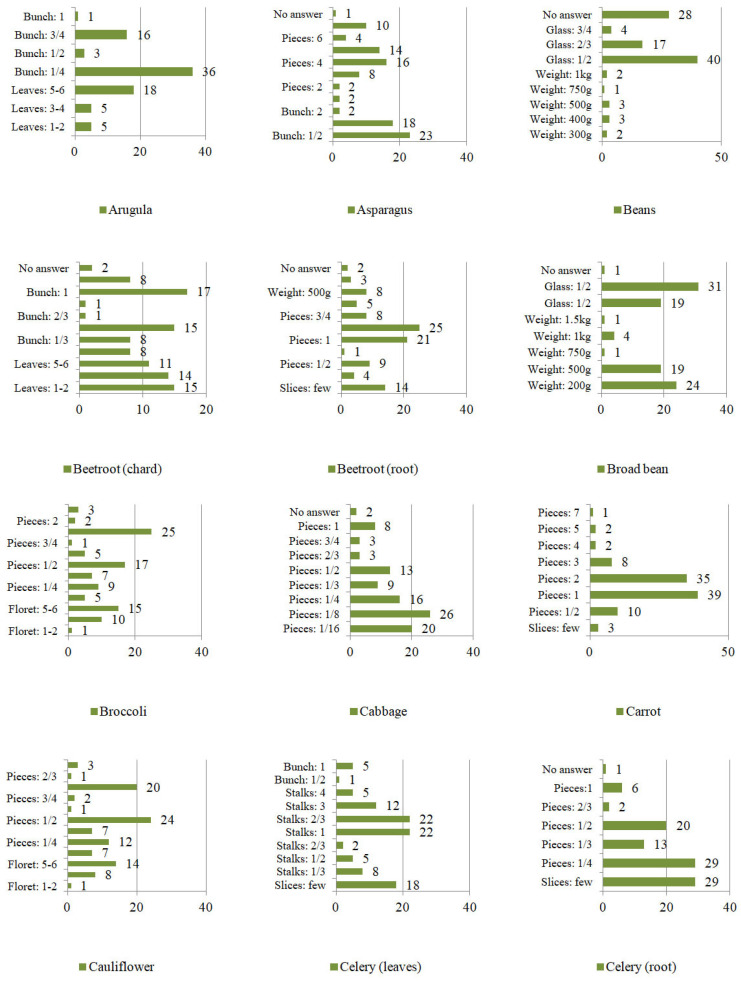
The quantity of vegetables consumed at one sitting by Poles [%].

**Figure 8:**
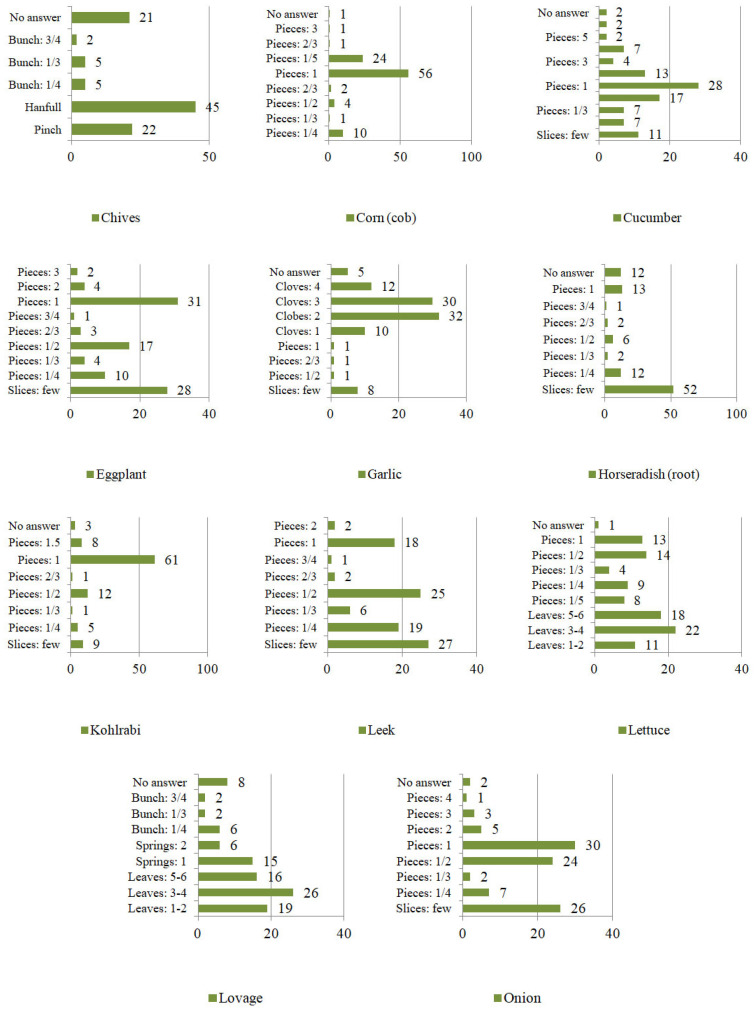
The quantity of vegetables consumed at one sitting by Poles [%] continued.

**Figure 9:**
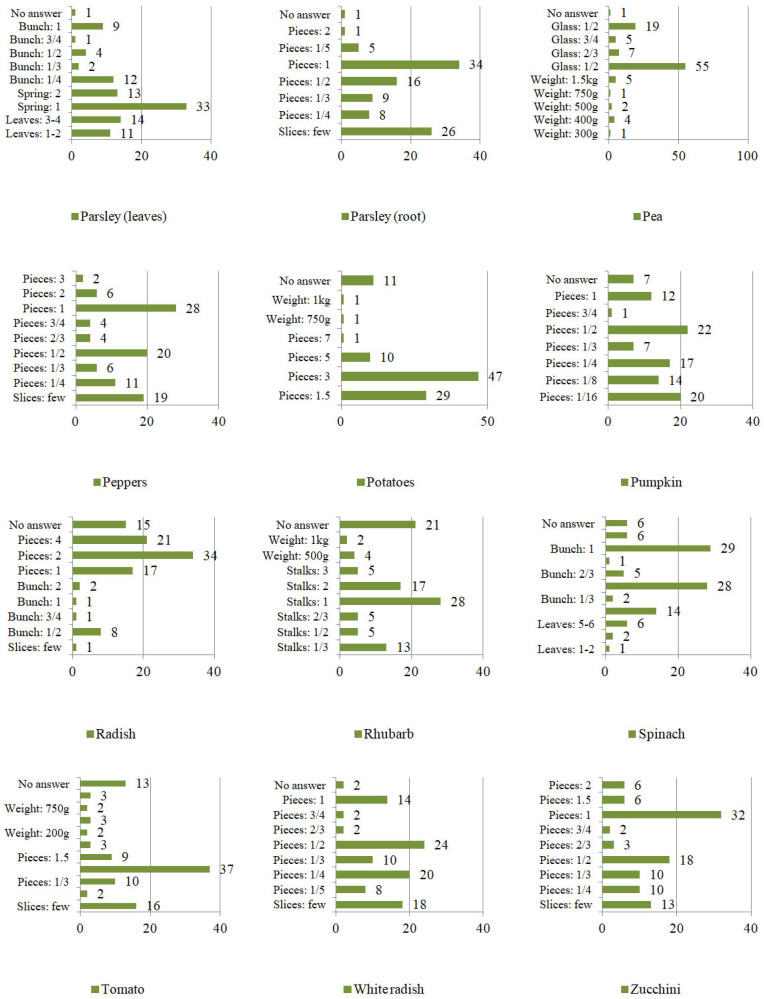
The quantity of vegetables consumed at one sitting by Poles [%] continued.

However, the frequency of eating was not the only element characterizing consumption patterns. The second variable taken into account was the quantity of vegetables consumed at one sitting (Figure 7, Figure 8 and Figure 9). The respondents specified the units of consumption of individual vegetables, not only by using weight units (g/km), but also the serving size (e.g., pieces, cloves, florets, leaves, stalks, twigs, bunches, a few slices, a pinch, a handful, a glass). Consumption was defined by units or the specific shape of the plant.

Mainly in the case of the following vegetables:

− leaves: beetroot, lovage, parsley, arugula, lettuce, and spinach;
− stalks: rhubarb, celery;
− twigs: lovage, parsley;
− a bunch: beetroot, lovage, parsley, arugula, radish, chives, asparagus, spinach;
− florets: broccoli, cauliflower;
− cloves: garlic;
− piece (e.g., head, tuber, root, shoot, cob): eggplant, broccoli, red beet, onion, horseradish, zucchini, garlic, pumpkin, cauliflower, kohlrabi, cabbage, corn, carrots, cucumbers, peppers, parsley (root), tomato, leek, radish, white radish, lettuce, celery, asparagus, potatoes.

On the other hand, approximate consumption concerned mainly the following vegetable plants:

− a pinch (of chopped vegetables): chives;
− a handful of (chopped vegetables): chives;
− a glass: broad beans, beans, and peas;
− slices: eggplant, beetroot, onion, horseradish, zucchini, garlic, kohlrabi, carrot, cucumbers, pepper, parsley (root), tomato, leek, radish, white radish and celery.

## 4. Discussion

Based on the survey on determining the preferences of Polish consumers in terms of buying vegetables either from conventional or organic farming and their dietary habits, the following general observations were revealed. Organic vegetables were chosen by 74% of the respondents. A total of 59% of them declared that they bought them ‘rarely’ or ‘sometimes’, and only 15% indicated they bought them ‘often’. The reasons for choosing certified organic vegetables included the willingness to eat healthy (30%) and the belief that organic vegetables are healthier (28%) or generally better (7%). The arguments against purchasing organic vegetables included no possibility of buying them close to the respondents’ residence (36%), high price (34%), and lack of actual advantages. In the last category of answers, the reasons most frequently indicated by the respondents were the lack of certainty about the essence of ecology (23%) and the fact that the respondents did not notice any significant differences between organic and non-organic vegetables (16%).

### 4.1. Vegetables from Conventional or Organic Farming?

Reducing the impact of consumption on the environment can be achieved by refraining from transporting food by air, limiting meat consumption, and choosing organic products [56]. Organic food is produced through farming practices that only use natural substances without the use of artificial fertilizers, herbicides, and pesticides, antibiotics, and genetically modified organisms (i.e., not exposed to irradiation). Moreover, it is grown using the process of crop rotation. The organic food sector is one of the fastest growing sectors of the food industry in the world. There are short- and long-term advantages of consuming such food. The short-term benefits include taste and satisfied curiosity. The long-term benefits include the nutritional value, a sense of security, improved health and environmental care [57] as well as the knowledge that no additional substances were used in their production [58].

Despite the discussed tendencies in organic farming, the conducted survey showed that respondents were rather not interested in organic food. Only one in ten people bought from an organic food store. Regardless of where organic vegetables were bought, more than half of the analyzed respondents used them rarely or sometimes. The respondents who took part in our surveys justified this decision by the fact that organic vegetables were difficult to buy, were not available close to their residence, or were too expensive. The reports on organic farming available in the literature have mainly discussed legal regulations related to agriculture/organic food in Poland, the number of organic farms, the area of organic farming, and the system of inspections carried out in a given year by these entities. Unfortunately, they do not indicate the possible places where vegetables can be purchased. The lack of actual benefits and the questionable transparency of certification procedures were also mentioned in our survey. This attitude may be caused by insufficient information on places offering organic products in particular cities and districts as well as the insufficient involvement of consumers in learning about certification and certification bodies.

The respondents who chose to buy organic products wanted to eat healthy (along with family members) and believed that organic vegetables were healthier and better. The aspect of organic production was emphasized only by one in 100 respondents. Similarly, the research conducted by Gustavsen [3] showed that prioritizing health was a stronger motivator to eat vegetables than prioritizing the pro-environmental goals.

Due to the above, it is recommended to not only continue informing the public about the importance of vegetables to a balanced diet, but also to promote information about places offering organic vegetables in Poland. In addition, it is also important to promote sustainable vegetable production as well as clearer visual information on the certification rules and the difference between specific organic and non-organic products.

The need for information has also been emphasized in other studies targeted at specific social groups (e.g., male consumers who are considered as less interested in healthy food than women). The combination of availability and nutritional information is an effective way to increase the consumption of vegetables without reducing food satisfaction [59].

### 4.2. Supermarkets or Fresh Food Markets?

Our survey showed that respondents most often bought vegetables at a supermarket. Nevertheless, shopping at the nearest supermarket was not always related to a geographical vicinity, but also influenced by other factors, especially when consumers travelled by car. Other elements included the economic factor (the price of a product), food preferences, habits, and cultural factors [60] as well as the possibility of shopping on the go to save time. Furthermore, supermarkets offer a large variety of products and not only local but also imported vegetables. However, the respondents who participated in the study were mainly interested in Polish vegetables, and only a few of them declared buying imported vegetables (sweet potatoes, bamboo shoots, and lemongrass). In terms of maintaining a balance between local and imported products, these choices may be perceived as balanced, but in terms of the diversity of consumed vegetables, they do not support a diverse diet. It is recommended by the Ministry of Funds and Regional Policy [61] to buy vegetables locally and seasonally. Polish society remains relatively ethnically homogeneous, as 95% of people living in Poland identify themselves as Poles, according to data provided by Statistics Poland [62]. Thus, importing vegetables is not as popular as in ethnically diverse societies such as those in Great Britain, France, or Germany. The choice of supermarkets as the main place for obtaining vegetables in Poland is not dictated by a wide range of products but by convenience (economic and time savings) and habits.

Second, according to the respondents, the most popular places to buy vegetables were those specializing in selling vegetables—so-called ‘greengrocers’, markets, and local shops. The availability of vegetables in the immediate vicinity of the place of residence encouraged the participants to buy them more frequently, especially those who were too busy working or did not own a means of transport and were less willing to shop far from home. Respondents in relationships, more often than single participants, shopped in local stores. Therefore, the local accessibility of vegetables may promote the consumption of the recommended servings and also increase their consumption in general [18].

### 4.3. Selling by Using Weight or Other Units?

Research evidence provides various methods to promote vegetables such as displaying them in more visible parts of stores, selling them with recipes, or during demonstrations on how to prepare vegetable dishes [63]. The conducted research indicated another method—portioning vegetables according to preferences. In Poland, vegetables are mostly bought raw, that is, either directly from the field, greenhouse, and garden, or after rinsing them, without any additional processing. The exception are frozen vegetables that are not only cleaned, but also peeled and cut. Fresh (not frozen) vegetables are usually sold by weight, by pieces, or in bundles. When several parts of the same plant may serve as food, they may be divided and sold separately (e.g., as root and parsley). The conducted research indicates that Polish consumers eat vegetables at once without paying attention to the weight of a given plant. However, they use units such as florets, slices, leaves, etc. Apart from vegetables, which are most often consumed cooked or combined (for example, chives or corn on the cob), they can be portioned in the same way as they are portioned at home to enhance buying vegetables as a snack. This concept may be supported by introducing reusable vegetable containers. This method would allow consumers to buy portions of vegetables—a few slices of kohlrabi, carrots, or cucumbers, a few leaves of lettuce or arugula. The use of such containers would also contribute to sustainable development through better meal planning and food waste prevention [64] as well as the eating of more vegetarian food [65].

Buying portioned vegetables will also allow consumers to try them on the go. When purchasing vegetables, consumers pay attention not only to their visual freshness and aroma, but also to their taste [66]. Taste is essential in the decision-making process [67], but it is effective only while tasting. Vegetables are rarely tasted before they are bought. Portioning them could change this. Globally, there is an increased demand for fresh-cut produce [68]. However, it has higher respiration rates than the corresponding intact products. Higher respiration rates indicate a faster deterioration rate as well as the danger of transmitting infectious diseases [68].

Regarding the questionnaire itself, it was revealed that a unified nomenclature is essential. In this type of survey, a clear definition was deemed to be important as there are discrepancies in classifying individual edible plants as vegetables or fruit. According to some definitions, vegetables are not fruits, while according to others, vegetables are superior not only to fruit, but also to roots, leaves, and inflorescences [69]. Unfortunately, this may affect the interpretation of the research results, especially in terms of proportions of the consumed food plants. Moreover, inconsistencies might appear not only considering if a plant belongs to the group of vegetables, but also if there are no differences in the name for the same plant across different regions of the country. This might have been a small issue here, as attempts to harmonize the nomenclature were implemented as much as possible before the questionnaire surveys were performed. Additionally, in the survey, the colloquial measure to determine the quantity of consumed vegetables was used such as cups, slices, etc. It helped and encouraged the respondents to finish the survey as most of them had probably never thought about how much each portion of various vegetables weighed and led to the fact that they were able to finish the survey before they became discouraged. However, this approach did not facilitate an easy recalculation for official measures such as grams or kilograms, which impoverish our observations.

The approach of collecting only one, the most suitable answer, from each respondent in the survey, differentiates our study significantly from similar questionnaire research projects. Such an attitude provided results that could be used for defining or calculating consumption patterns, for instance, among subpopulations and specified vegetables. This type of information is currently missing in national statistical research surveys.

### 4.4. Limitations

Our research constituted 108 complete surveys that were further investigated in the project, thus it might have been the main limitation of the study. Among the respondents, 74% were women, which could affect the underestimation of the statistical mean value of vegetable consumption, as women consumed more vegetables than men [70]. On the other hand, as women were mainly responsible for supplying the household with food, they had a significant impact on what was consumed, which could affect the increase in consumed vegetables, definitely by children and probably also by men. The majority of respondents were between 21 and 50 years old (75%). This might also affect the statistical mean consumption rate as practically, children and adolescents, on one hand, and seniors on the other, were not included sufficiently in the research. However, our results might be considered relevant to determine the vegetable consumption rates and preferences among the so-called ‘statistical Poles of working age’. Furthermore, among our respondents, most of them possessed a higher education level (73%), which could correspond to relatively high awareness and knowledge of a healthy lifestyle and nutrition [71]. The declared level of education among respondents also corresponded quite well with the declared income of most of the respondents. The respondents’ declared income was between PLN1000 and PLN3000 (US$251.7–754.5; 52%), which corresponded quite well with the earnings to be able to afford (the economical possibility) better food including vegetables. Additionally, 92% declared that living in urban areas might have affected the high awareness of healthy eating [72]. The limited number of the completed surveys that were gathered may also have influenced not finding other significant relationships between consumption patterns and the various socio-demographic groups.

## 5. Conclusions

The conducted survey on consumption habits and motivation for choosing vegetables grown in Poland offered considerable insights into sustainable vegetable consumption in Poland and contributed to a better understanding of consumer behavior toward organic vegetables. Our research revealed that the majority of respondents (74%) declared consuming organic vegetables, however, most of them did so occasionally. According to the respondents’ main reasons for consuming organic vegetables were those related to their positive impact on health. Among the reasons for not consuming organic vegetables declared by the respondents were the lack of access to them at the place of residence, high price, and the belief of the lack of their advantages. The study was also an introduction and a base point for further in-depth research, both in terms of the characteristics of the respondents and the discussed vegetables. The results of this study may be useful in further social, sociological, and environmental research, particularly in the assessment of health and environmental risks due to the precise and systematic structure of consumption and dietary exposure including a wider group of respondents and more detailed characteristics of vegetables.

## Figures and Tables

**Table 1:** Description of the vegetables investigated in this study.

No.	Common Name	Botanical Name
1.	Arugula	Eruca sativa
2.	Asparagus	Asparagus officinalis
3.	Aubergine/Eggplant	Solanum melongena
4.	Beans	Phaseolus
5.	Beetroot	Beta vulgaris
6.	Broccoli	Brassica oleracea
7.	Broad beans	Vicia faba
8.	Brussels sprouts	var. gemmifera
9.	Cauliflower	Brassica oleracea var. botrytis
10.	Cabbage	Brassica oleracea var. botrytis
11.	Carrot	Daucus carota
12–13.	Celery root/celery	Apium graveolens var. rapaceum/Apium graveolens
14.	Chives	Allium schoenoprasum
15.	Corn	Zea mays
16.	Cucumber	Cucumis sativus
17.	Garlic	Allium sativum
18.	Horseradish	Armoracia rusticana
19.	Kohlrabi	Brassica oleracea var. gongylodes
20.	Leek	Allium porrum
21.	Lettuce	Lactuca sativa
22.	Lovage	Armoracia rusticana
23.	Onion	Allium cepa
24–25.	Parsley tops/Parsley root	Petroselinum hortense Capsicum annuum
26.	Pea	Pisum sativum
27.	Potato	Solanum tuberosum
28.	Pumpkin	Cucurbita pepo
29.	Radish	Raphanus sativus var. sativus
30.	Rhubarb	Rheum rhaponticum
31.	Spinach	Brassica oleracea
32–34.	Sweet pepper/Ground pepper/Hot pepper	Capsicum annuum/Piper nigrum/Capsicum frutescens
35.	Tomato	Lycopersicon esculentum
36.	Zucchini/Squash	Cucurbita pepo convar. giromontiina greb

**Table 2:** The socio-demographic characteristics of Polish respondents.

	Demographic Factor	Frequency (n = 108)	Percentage (%)
Gender	Male	29	27
	Female	79	73
	Prefer not to answer	0	0
Age	18–20 years	2	2
	21–30 years	24	22
	31–40 years	44	41
	41–50 years	13	12
	51–60 years	9	8
	61–70 years	10	9
	Over 70 years	6	6
Educational level	Secondary education	7	7
	Secondary vocational	2	2
	Post-secondary	9	8
	Higher vocational	4	4
	Bachelor degree	9	8
	Master degree	73	68
	Other	3	3
Marital status	Single	31	29
	Married/in relation	64	59
	Separation/after divorce	4	4
	Widowed	5	5
	Refusal to answer	4	3
Region of Poland (voivodeship/ province)	Dolnośląskie	14	13
	Kujawsko-Pomorskie	1	1
	Lubuskie	2	2
	Łódzkie	20	19
	Małopolskie	24	22
	Opolskie	27	25
	Podkarpackie	1	1
	Pomorskie	1	1
	Śląskie	5	5
	Warmińsko-Mazurskie	4	4
	Wielkopolskie	3	3
	Zachodniopomorskie	4	4
Area of residence, number of inhabitants	Countryside, agricultural area	9	8
	Countryside, industrialized area	3	3
	City, up to 20,000	5	5
	City, 21,000–100,000	8	7
	City, 101,000–250,000	20	19
	City, 251,000–500,000	10	9
	City, 501,000–750,000	9	8
	City, 751,000–1,000,000	24	22
	City, over 1,000,000	16	15
	Refusal to answer	4	4
Number of people in the household	1	16	15
	2	47	44
	3	15	14
	4	19	18
	5	5	5
	6	1	1
	7	1	1
	8	1	1
	Refusal to answer	3	3
Indicative net income in PLN [in USD]	Up to PLN1000 [US$251.5]	3	3
	PLN1001–3000 [US$251.7–754.5]	56	52
	PLN3001–5000 [US$754.8–1257.6]	16	15
	PLN5001–7000 [US$1257.8–1760.6]	13	12
	PLN7001–9000 [US$1760.8–2263.6]	3	3
	Over PLN9000 [US$2263.6]	2	2
	Refusal to answer	15	14

**Table 3:** The frequency of vegetable intake grown in Poland [%].

**Vegetable**	**The Frequency of Consumption [%] (Number of Times in a Given Unit of Time)**												
	**Daily**			**Weekly**						**Monthly**	**Annually**		**Not** **at All**
	**3**	**2**	**1**	**6**	**5**	**4**	**3**	**2**	**1**	**Few**	**Dozen**	**Few**	
Eggplant	-	1	-	-	-	1	-	1	3	12	20	39	23
Broccoli	-	-	2	-	-	2	3	9	16	34	24	7	3
Beetroot (* root/chard)	-	-	-	-	-	2/-	2/-	4/2	13/28	34/-	25/-	16/-	5/4
Broad bean	-	-	2	-	-	1	8	6	27	-	-	-	26
Onion	-	4	18	3	4	16	11	18	10	9	4	2	1
Horseradish	-	-	-	-	1	-	1	2	4	16	22	35	19
Zucchini	-	-	2	-	-	3	5	4	11	19	30	18	6
Garlic	-	-	7	3	4	6	12	13	13	18	9	7	6
Pumpkin	-	-	2	-	-	3	4	4	31	-	-	-	37
Beans	-	-	-	1	1	3	-	4	6	19	33	25	8
Pea	-	-	-	-	-	-	1	1	2	12	29	35	20
Cauliflower	-	-	-	-	1	1	2	4	9	36	36	7	3
Kohlrabi	-	-	2	-	1	1	7	13	21	-	-	-	39
Cabbage	-	-	3	2	-	3	4	7	12	38	20	6	3
Corn (cob)	-	-	2	-	1	2	5	8	30	-	-	-	24
Lovage	-	-	1	-	1	1	1	5	6	10	16	18	42
Carrot	-	2	8	6	6	10	23	10	16	18	1	-	-
Cucumber	-	3	4	3	3	6	10	14	20	21	11	2	1
Peppers	-	3	2	3	2	5	9	13	9	33	10	6	5
Parsley (* root/leaves)	-/1	-	2/4	3/3	½	4/8	8/8	13/12	19/12	19/25	15/15	10/3	6/6
Tomato	3	8	10	4	3	16	13	15	7	11	4	-	4
Leek	-	1	-	-	1	2	2	5	13	29	22	17	9
Rhubarb	1	-	1	-	-	-	3	4	23	-	-	-	51
Arugula	-	1	-	2	-	3	3	6	5	21	21	20	18
Radish	-	2	4	1	1	2	6	6	9	21	32	10	4
White radish	-	-	-	1	-	-	-	1	2	4	18	20	54
Lettuce	-	1	4	2	6	3	12	9	17	18	17	7	4
Celery (* root/leaves)	-	-/1	1/-	-	1/-	1/-	6/-	10/2	13/1	20/13	16/19	17/20	16/44
Chives	-	3	2	-	2	4	4	8	14	21	29	6	6
Asparagus	-	-	2	-	-	-	4	9	15	-	-	-	53
Spinach	-	-	-	-	1	5	8	11	31	-	-	-	25
Potatoes	-	1	6	6	5	14	13	19	13	16	4	2	1

* For example, for the vegetable beetroot (a: root/b: chard or a: root/ b: leaves), the numerical value is written as a/b.

## Data Availability

Not applicable.

## References

[1] Idowu S.O. (2012). Encyclopedia of Corporate Social Responsibility.

[2] Fischer C.G., Garnett T. (2016). Plates, Pyramids, Planet Developments in National Healthy and Sustainable Dietary Guidelines: A State of Play Assessment. Food and Agriculture Organization of the United Nations.

[3] Gustavsen G.W. (2020). Motivations for Sustainable Consumption: The Case of Vegetables. Int. J. Food System Dyn..

[4] Murawska A. (2016). Changes in Vegetable Consumption in Poland in the Context of Sustainable Consumption, (in Polish: Zmiany w Spożyciu Warzyw w Polsce w Kontekście Zrównoważonej Konsumpcji). Rocz. Nauk. Stowarzyszenia Ekon. Rol. I Agrobiz..

[5] Veerman C., Pinto Correia T., Bastioli C., Biro B., Bouma J., Cienciala E., Emmett B., Frison E.A., Grand A., Filchew L.H. (2020). Caring for Soil Is Caring for Life—Ensure 75% of Soils Are Healthy by 2030 for Food, People, Nature and Climate.

[6] World Health Organisation (2003). Diet, Nutrition and the Prevention of Chronic Diseases, Report of a Joint WHO/FAO Expert Consultation, WHO Technical Report Series 916.

[7] Ramya V., Priya P. (2019). Health benefits of vegetables. Int. J. Chem. Stud..

[8] Silva Dias J.C., Imai S. (2017). Vegetables Consumption and its Benefits on Diabetes. J. Nutr. Ther..

[9] Hurtado-Barroso S., Trius-Soler M., Lamuela-Raventos R.M., Zamora-Ros R. (2020). Vegetable and Fruit Consumption and Prognosis Among Cancer Survivors: A Systematic Review and Meta-Analysis of Cohort Studies. Adv. Nutr..

[10] Villegas R., Shu X.O., Gao Y.-T., Yang G., Elasy T., Li H.L., Zheng W. (2008). Vegetable but Not Fruit Consumption Reduces the Risk of Type 2 Diabetes in Chinese Women. J. Nutr. Nutr. Epidemiol..

[11] Broers V.J.V., Van den Broucke S., Luminet O. (2020). Determinants of prebiotic vegetable consumption: The extended theory of planned behavior. Arch. Public Health.

[12] Carrillo J.A., Zafrilla M.P., Marhuenda J. (2019). Cognitive Function and Consumption of Fruit and Vegetable Polyphenols in a Young Population: Is There a Relationship?. Foods.

[13] Kołota A., Głąbska D. (2021). Analysis of Food Habits during Pandemic in a Polish Population-Based Sample of Primary School Adolescents: Diet and Activity of Youth during COVID-19 (DAY-19) Study. Nutrients.

[14] Noorwali E.A.A. (2019). Sleep and Fruit and Vegetable Consumption in UK Adults. Ph.D. Thesis.

[15] Cheng H.-Y., Shi Y.-X., Yu F.-N., Zhao H.-Z., Zhang J.-H., Song M. (2019). Association between vegetables and fruits consumption and depressive symptoms in a middle-aged Chinese population. An observational study. Medicine.

[16] Jarosz M. (2017). Nutrition Standards for the Polish Population, (In Polish: Normy Żywienia dla Populacji Polski).

[17] Motkuri V. (2020). Vegetable Consumption in India: Supply Chain and Prices.

[18] Xaba T., Dlamini S. (2021). Factors associated with consumption of fruits and vegetables amongst adults in the Alfred Duma Local Municipality, Ladysmith. S. Afr. J. Clin. Nutr..

[19] Siregar A., Krisnasary A., Simbolon D. (2021). Differences of Fruit-Vegetable Consumption, Blood Pressure in Highland And Lowland. J. Ilmu Dan Teknol. Kesehat..

[20] Olatona F.A., Sosanya A., Sholeye O.O., Obrutu O.E., Nnoaham K.E. (2018). Knowledge of fruits and vegetables, consumption pattern and associated factors among adults in Lagos State, Nigeria. Res. J. Health Sci..

[21] Johnson J.S., Nobmann E.D., Asay E. (2012). Factors related to fruit, vegetable and traditional food consumption which may affect health among Alaska Native People in Western Alaska. Int. J. Circumpolar Health.

[22] Vitale M., Giosuè A., Vaccaro O., Riccardi G. (2021). Recent Trends in Dietary Habits of the Italian Population: Potential Impact on Health and the Environment. Nutrients.

[23] Dietetycy.org.pl.

[24] www.portalspozywczy.pl. In 2020, Poles Spent More on Fruit and Vegetables (In Polish: W 2020 r. Polacy Więcej Wydali na Owoce i Warzywa). https://www.portalspozywczy.pl/napoje/wiadomosci/w-2020-r-polacy-wiecej-wydali-na-owoce-i-warzywa,200308.html.

[25] Hall J.N., Moore S., Harper S.B., Lynch J.W. (2009). Global Variability in Fruit and Vegetable Consumption. Am. J. Prev. Med..

[26] GUS (2020). Household Budget Survey in 2019.

[27] James D.C.S. (2004). Factors Influencing Food Choices, Dietary Intake, and Nutrition-Related Attitudes among African Americans: Application of a Culturally Sensitive Model. Ethn. Health.

[28] Lima J.P.M., Costa S.A., Brandao T.R.S., Rocha A. (2021). Food Consumption Determinants and Barriers for Healthy Eating at the Workplace—A University Setting. Foods.

[29] Turnbull O., Homer M., Ensaff H. (2021). Food insecurity: Its prevalence and relationship to fruit and vegetable consumption. J. Hum. Nutr. Diet..

[30] GUS (2011). Sustainable Development Indicators for Poland [in Polish: Wskaźniki Zrównoważonego Rozwoju Polski].

[31] Inspekcja Skupu i Przetwórstwa Artykułów Rolnych (2001). Organic Farming in Poland in the Years 1999—2000 (in Polish: Rolnictwo Ekologiczne w Polsce w Latach 1999—2000).

[32] Inspekcja Skupu i Przetwórstwa Artykułów Rolnych (2002). Agricultural Production with Ecological Methods in 2001 (in Polish: Produkcja Rolna Metodami Ekologicznymi w 2001 Roku).

[33] Inspekcja Skupu i Przetwórstwa Artykułów Rolnych (2003). Organic Farming in Poland in 2002 (in Polish: Rolnictwo Ekologiczne w Polsce w 2002 Roku).

[34] IJHARS (2004). Organic Farming in Poland in 2003 (in Polish: Rolnictwo Ekologiczne w Polsce w 2003 Roku).

[35] IJHARS (2005). Organic Farming in Poland in 2004 (in Polish: Rolnictwo Ekologiczne w Polsce w 2004 Roku).

[36] IJHARS (2007). Condition of Organic Farming in Poland. The Report 2005–2006 (in Polish: Raport o Stanie Rolnictwa Ekologicznego w Polsce w Latach 2005–2006).

[37] IJHARS (2009). Condition of Organic Farming in Poland. The Report 2007–2008 (in Polish: Raport o Stanie Rolnictwa Ekologicznego w Polsce w Latach 2007–2008).

[38] IJHARS (2011). Condition of Organic Farming in Poland. The Report 2009–2010 (in Polish: Raport o Stanie Rolnictwa Ekologicznego w Polsce w Latach 2009–2010).

[39] IJHARS (2013). Condition of Organic Farming in Poland. The Report 2011–2012 (in Polish: Raport o Stanie Rolnictwa Ekologicznego w Polsce w Latach 2011–2012).

[40] IJHARS (2015). Condition of Organic Farming in Poland. The Report 2013–2014 (in Polish: Raport o Stanie Rolnictwa Ekologicznego w Polsce w Latach 2013–2014).

[41] IJHARS (2017). The Report on Organic Farming in Poland in 2015–2016 (in Polish: Raport o Stanie Rolnictwa Ekologicznego w Polsce w Latach 616 2015–2016).

[42] IJHARS (2019). The Report on Organic Farming in Poland in 2017–2018 (in Polish: Raport o Stanie Rolnictwa Ekologicznego w Polsce w Latach 619 2017–2018).

[43] IJHARS (2021). The Report on Organic Farming in Poland in 2019–2020 (in Polish: Raport o Stanie Rolnictwa Ekologicznego w Polsce w Latach 2019–2020).

[44] Krakowy Ośrodek Wsparcia Rolnictwa (2018). Vegetable Market in Poland (in Polish: Rynek Warzyw w Polsce).

[45] Jąder K., Wawrzyniak J. (2015). Changes in the Consumption of Fruits and Vegetables and their Preserves in Poland in 1999‒2013 and the Phenomenon of Sustainable Consumption (in Polish: Zmiany w Spożyciu Owoców i Warzyw Oraz ich Przetworów w Polsce w Latach 1999‒2013 a Zjawisko Zrównoważonej Konsumpcji). J. Agribus. Rural. Dev..

[46] Groele B., Głąbska D., Gutkowska K., Guzek D. (2019). Mothers’ Vegetable Consumption Behaviors and Preferences as Factors Limiting the Possibility of Increasing Vegetable Consumption in Children in a National Sample of Polish and Romanian Respondents. Nutrients.

[47] Harton A., Florczak J., Myszkowska-Ryciak J., Gajewska D. (2015). Fruit and vegetable consumption by preschool children (in Polish: Spożycie warzyw i owoców przez dzieci w wieku przedszkolnym). Probl. Hig. Epidemiol..

[48] Malczyk E., Całyniuk Z., Syc M. (2016). Assessment of the Frequency of Consumption of Fruits and Vegetables by Students of Medical University of Lublin (in Polish: Ocena czystości spożycia warzyw i owoców przez studentów Uniwersytetu Medycznego w Lublinie). Bromat. Chem. Tosykol.

[49] Szmidt M., Granda D., Broda A., Brzozowska A. (2019). The Role of Vegetables and Fruits in the Diet of the Eldery (in Polish: Rola warzyw i owoców w diecie osób starszych). KOSMOS Probl. Nauk. Biol..

[50] KANTAR (2020). Vegetables and Fruit in the Diet of Poles. National Fruit and Vegetable Consumption Survey, (in Polish: Warzywa i Owoce w Diecie Polaków. Narodowe Badania Konsumpcji Warzyw i Owoców).

[51] Kawalec A., Pawlas K. (2021). Breakfast Frequency and Composition in a Group of Polish Children Aged 7–10 Years. Nutrients.

[52] Skolmowska D., Głąbska D., Guzek D. (2021). Association between Food Preferences and Food Habits in a Polish Adolescents’ COVID-19 Experience (PLACE-19) Study. Nutrients.

[53] Gheribi E. (2012). Consumption of fruit and vegetable in Polish households in the period of 2004–2008 (in Polish: Konsumpcja owoców i warzyw w polskich gospodarstwach domowych w latach 2004–2008). Ekonomika i Organizacja Gospodarki Żywnościowej.

[54] Suliga E., Cieśla W., Michel S., Kaducakova H., Martin T., Sliwiński G., Braun A., Izova M., Lehotska M., Kozieł D. (2020). Diet Quality Compared to the Nutritional Knowledge of Polish, German, and Slovakian University Students—Preliminary Research. Int. J. Environ. Res. Public Health.

[55] Questionpro, Snowball Sampling: Definition, Method, Advantages and Disadvantages. https://www.questionpro.com/blog/snowball-sampling/.

[56] Onwezen M.C. (2015). I did good, and we did bad: The impact of collective versus private emotions on pro-environmental food consumption. Food Res. Int..

[57] Mohsen M.G., Dacko S. (2013). An extension of the benefit segmentation base for the consumption of organic foods: A time perspective. J. Mark. Manag..

[58] Wojciechowska-Solis J., Barska A. (2021). Exploring the Preferences of Consumers’ Organic Products in Aspects of Sustainable Consumption: The Case of the Polish Consumer. Agriculture.

[59] Uglem S., Stea T.H., Kjollesdal M.K.R., Frolich W., Wandel M. (2013). A nutrition intervention with a main focus on vegetables and bread consumption among young men in the Norwegian National Guard. Food Nutr. Res..

[60] Aggarwal A., Cook A.J., Jiao J.F., Seguin R.A., Moudon A.V., Hurvitz P.M., Drewnowski A. (2014). Access to Supermarkets and Fruit and Vegetable Consumption. Am. J. Public Health.

[61] Ministerstwo funduszy i polityki regionalnej (2019). Responsible Consumer (in Polish: Odpowiedzialny Konsument).

[62] GUS (2015). National-Ethnic, Linguistic and Religious Structure of the Polish Population. 2011 National Census of Population and Housing (in Polish: Struktura Narodowo-Etniczna, Językowa i Wyznaniowa Ludności Polski. Narodowy Spis Powszechny Ludności i Mieszkań 2011).

[63] MacLellan D.L., Gottschall-Pass K., Larsen R. (2004). Fruit and Vegetable Consumption: Benefits and Barriers. Can. J. Diet. Pract. Res..

[64] Fuentes C., Samsioe E. (2021). Devising food consumption: Complex households and the socio-material work of meal box schemes. Consum. Mark. Cult..

[65] Rahman S., Luomala H. (2020). A Comparison of Motivational Patterns in Sustainable Food Consumption between Pakistan and Finland: Duties or Self-Reliance?. J. Int. Food Agribus. Mark..

[66] Kowrygo B., Sawicka B., Świstak E. (1997). Changes in Fruit and Vegetables Consumption in Poland during 1990–1995 (in Polish: Zmiany w spożyciu owoców i warzyw w Polsce w latach 90). Żywność. Technologia. Jakość.

[67] Gelinder L., Hjälmeskog K., Lidar M. (2020). Sustainable food choices? A study of students’ actions in a home and consumer studies classroom. Environ. Educ. Res..

[68] Qadri O.S., Yousuf B., Srivastava A.K. (2015). Fresh-cut fruits and vegetables: Critical factors influencing microbiology and novel approaches to prevent microbial risks—A review. Cogent Food Agric..

[69] PWN Polish Language Dictionary [In Polish: Słownik Języka Polskiego]. https://sjp.pwn.pl/szukaj/warzywa.html.

[70] Masella R., Malorni W. (2017). Gender-related differences in dietary habits. Clin. Manag. Issues.

[71] Raghupathi V., Raghupathi W. (2020). The influence of education on health: An empirical assessment of OECD countries for the period 1995–2015. Arch. Public Health.

[72] Fard N.A., Morales G.D.F., Mejova Y., Schifanella R. (2021). On the interplay between educational attainment and nutrition: A spatially-aware perspective. EPJ Data Sci..

